# Macrogenomic analysis showcases the diversity of tick RNA viruses in Mentougou, Beijing, China

**DOI:** 10.1016/j.nmni.2026.101819

**Published:** 2026-07-24

**Authors:** Qiangqiang Shi, Qinqin Song, Xuan Liu, Guoyong Mei, Chen Gao, Haijun Du, Zhiqiang Xia, Mi Liu, Juan Song, Lifeng Zhang, Rundong Zhu, Ze Cheng, Jin Cao, Duo Rao, Yuqin Zhang, Zhiyue Wang, Jun Han

**Affiliations:** a National Key Laboratory of Intelligent Tracking and Forecasting for Infectious Diseases, National Institute for Viral Disease Control and Prevention, Chinese Center for Disease Control and Prevention & Chinese Academy of Preventive Medicine, Beijing, China; b Mentougou Center for Disease Control and Prevention, Beijing, China

**Keywords:** Viral diversity, Meta-transcriptome, Tick-borne virus, Beijing

## Abstract

**Background:**

Ticks are the second most significant vector of human pathogens worldwide, with 911 documented species globally and a broad distributed across China. Currently, over 160 tick-borne viruses (TBVs) have been identified, several of which pose substantial threats to human health, such as Dabie bandavirus, Jingmen tick virus, Alongshan virus, Songling virus, Beiji nairovirus, and Langya henipavirus, raising increasing global attention. Despite their significance, the diversity of TBVs in Beijing remains poorly characterized.

**Methods:**

In this study, we conducted metagenomic sequencing on tick samples collected from Mentougou District, Beijing. The obtained reads were subjected to quality control, de novo assembly, and viral sequence identification, followed by phylogenetic and evolutionary analyses.

**Results:**

Our results identified 19 distinct viral species spanning 12 families, including *Hepelivirales, Solemoviridae,Mymonaviridae, Nodaviridae,Permutotetraviridae, Phenuiviridae,Rhabdoviridae, Tombusviridae,Totiviridae, Peribunyaviridae,Flaviviridae, Nodaviridae,Tymoviridae, Tombusviridae*. Among these, six novel viruses from five virus families were discovered. A potential pathogen, Tick jingmen-like virus, was also detected in the selected pathogens. These findings underscore the remarkable diversity of RNA viruses harbored by ticks in Mentougou District.

**Conclusions:**

Our research findings reveal a previously unrecognized diversity of tick-borne viruses in the Mentougou District, Beijing, and provide essential baseline data for informing future surveillance strategies and guiding prevention and control of tick-borne diseases in the Beijing metropolitan area.

## 1 Introduction

Ticks are blood-sucking arthropods belonging to order *Acarina*, class *Arachnida*, family *Ixodidae* [1]. Of the 911 recognized tick species globally, 709 belong to the highly diverse Ixodidae family. [2]. As major vectors of human and zoonotic pathogens, ticks transmit infectious agents during their four-stage life cycle (egg, larva, nymph, and adult), which involves prolonged attachment to hosts for 4–14 days [3]. This feeding behavior facilitates the transmission of various pathogens to mammals, reptiles, amphibians, and birds [4]. Medically significant TBVs primarily fall within the families *Flaviviridae* and *Nairoviridae*, including West Nile virus, Crimean-Congo hemorrhagic fever virus (CCHFV), tick-borne encephalitis virus (TBEV), and Dabie bandavirus [2]. The spread of these viruses has led to significant morbidity and mortality, highlighting their public health impact. [[5], [6], [7]]. For instance, 10,000 to 15,000 infection cases of CCHFV are reported worldwide annually. In recognition of its considerable medical threat, the World Health Organization (WHO) designated it as a priority pathogen in December 2015 [8]. Most TBVs are RNA viruses, characterized by high mutation rates that facilitate adaptation through host interactions. Although tick RNA viromes have been studied in neighboring regions such as Inner Mongolia, data from Beijing remain limited [9,10].

Next-generation sequencing (NGS) is a high-throughput and efficient method for nucleic acid sequencing that relies on polymerase chain reaction (PCR) and gene chips [11]. Since its initial application to assess the diversity of tick bacterial composition in 2011, a total of 683 tick-borne viruses (TBVs) have been identified using NGS technology [2,12]. Notably, in 2023, Cao et al. reported the identification of 1801 RNA virus genomes across 31 tick species, which included a total of 724 viruses, 501 of which were previously unrecognized [13]. These findings substantially enhance the existing virus database, reshape our understanding of the tick-borne virus spectrum, and provide critical background data for the early detection of emerging infectious diseases.

As an international metropolis in northern China, Beijing's rapid urbanization and high population density may heighten the risk of tick transmission. Previous studies have examined tick RNA viromes in Beijing and its neighboring regions, including Inner Mongolia, Hebei, and Tianjin [9,14,15]. However, the specific distribution of viruses across various districts of Beijing remains inadequately explored. Consequently, this study employed NGS metagenomics technology to analyze tick samples from the Mentougou area. The objective was to comprehensively assess the diversity, molecular evolutionary characteristics, and potential public health risks associated with tick-borne viruses in the region. Our findings are expected to provide a scientific basis for the local prevention and control of tick-borne diseases, fill critical gaps in the regional virus background data, and contribute valuable information to the global tick-borne virus database.

## 2 Materials and methods

### 2.1 Sample collection and identification

**Fig. 1:**
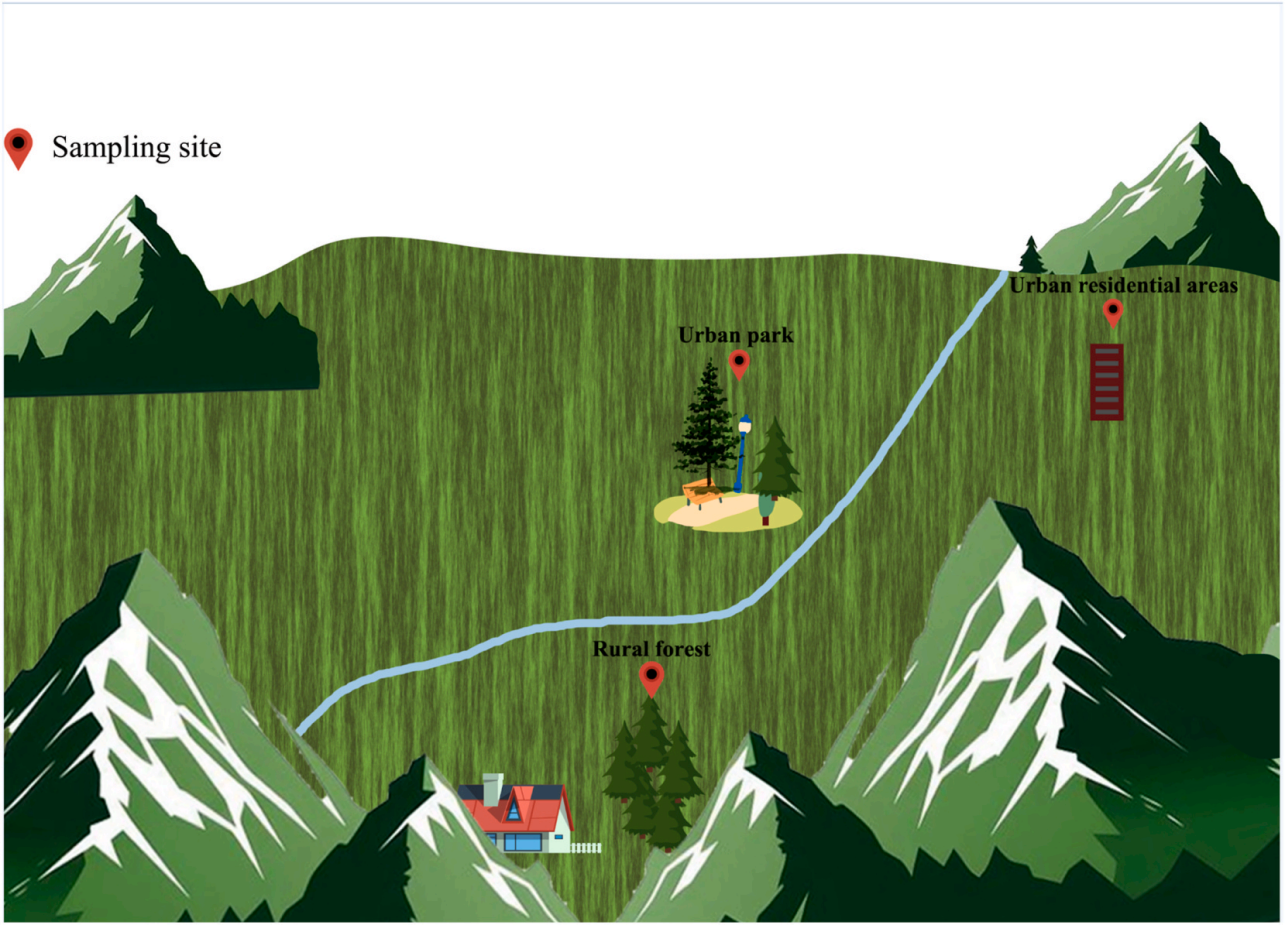
_Schematic diagram of sampling sites. Three sampling points were selected based on their geographical location and ecological environment, with each sampling point indicating its ecological environment.

In July 2024, host-seeking ticks were collected from three sampling sites in Mentougou District, Beijing, China (Fig. 1). The sites were selected to represent three distinct ecological landscapes: an urban residential area (Site 1: 116.09°E, 40.00°N), an urban park ecosystem (Site 2: 116.10°E, 39.98°N), and a rural forest ecosystem (Site 3: 116.06°E, 39.99°N). At each site, ticks were collected using the standard flagging method (Table S1). Ticks were pooled by sampling site and collection date, placed in sterile 2 mL cryogenic vials, and immediately stored in a portable liquid nitrogen dry shipper. Upon arrival at the laboratory, specimens were stored at −80 °C until further processing. Morphological identification of tick species was performed under a stereomicroscope using standard taxonomic keys. Representative specimens from each site were further confirmed by PCR amplification of the mitochondrial 16S rRNA gene (F:5′-GCTGCTCAATGATTTTTTAAATTGCTGTGGG-3′, R: 5′-CCGGGTCTGAACTCAGATCAAGT-3′) [16].

### 2.2 RNA library construction and second-generation sequencing

Samples were washed with Phosphate Buffered Saline (PBS, HyClone, Logan, UT, USA; Catalog No.: SH30256.01), homogenized in Dulbecco's Modified Eagle Medium (DMEM, GIBCO, Waltham, MA, USA; Catalog No.: 11995065), and centrifuged. Total RNA was extracted using TRIzol reagent (Thermo Fisher Scientific, Waltham, MA, USA; Catalog No.: 1199506515596026CN), quantified via Qubit 4 Fluorometer and NanoDrop™ One (Thermo Fisher Scientific, Waltham, MA, USA; Catalog No.: 840-317500), and ribosomal RNA was depleted. Libraries were prepared using the MGI Easy Fast RNA Library Preparation Kit (MGI Tech, Shenzhen, Guangdong, China; Catalog No.: 940-002921-00) and sequenced on the MGISEQ-2000 platform (100 bp paired-end; MGI Tech, Shenzhen, Guangdong, China).

### 2.3 Data cleaning and assembly

Raw reads were quality-controlled with Fastp v. 0.20.1, and host/rRNA sequences were removed using SortMeRNA v. 4.3.6 and Bowtie2 v. 2.4.4. *De novo*assembly was performed with SPAdes v. 3.15.5, and contigs were annotated via blastx v. 2.14.0 against the NCBI NR database. Viral sequences were classified as novel if they exhibited <80% nucleotide identity over the full genome or <90% amino acid identity in the RNA-dependent RNA polymerase (RdRp) domain [17,18].

### 2.4 Sequence analysis

For the protein sequence of TJMV, we predicted its transmembrane region (TMHMM 2.0: www.cbs.dtu.dk/services/TMHMM/), signal peptide region (SignalP 6.0: https://services.healthtech.dtu.dk/services/SignalP-6.0/), and N-linked glycosylation sites (NetNGlyc 1.0: https://services.healthtech.dtu.dk/services/NetNGlyc-1.0/). Simultaneously predict the secondary structure (InterPro: https://www.ebi.ac.uk/interpro/). All open reading frames (ORFs) of the sequences were obtained through ORFfinder (https://www.ncbi.nlm.nih.gov/orffinder/), and the proteins encoded by ORFs were obtained through CD-Search (https://www.ncbi.nlm.nih.gov/Structure//cdd/wrpsb.cgi). For TJMV, we also predicted its B cell (ABCpred: https://webs.iiitd.edu.in/raghava/abcpred/ABC_submission)and T cell epitopes (NetMHCIIpan: https://services.healthtech.dtu.dk/services/NetMHCIIpan-4.0).

### 2.5 Phylogenetic analysis

Phylogenetic trees were constructed using IQ-tree3 with 1000 bootstrap replicates. For TJMV, protein domains were predicted using TMHMM, SignalP, NetNGlyc, and InterPro. Align the amino acid sequences of RdRp and the ORF containing the polyprotein in the virus sequence with the relevant reference sequences downloaded from GenBank using MEGA7's MUSCLE package and default parameters. Save the comparison results in FASTA format. Construct a maximum likelihood (ML) phylogenetic tree using the IQ-tree3 algorithm, perform optimal model adaptation based on sequence alignment, and perform fast bootstrap with 1000 repetitions.

## 3 Results

### 3.1 Virus identification

About 120 ticks belonging to the genus *Haemaphysalis* of the family *Ixodidae* were collected from three sampling sites. Eight RNA libraries generated approximately 24 Gb of clean data, resulting in the identification of 300 viral sequences. Among these, complete or near-complete genome sequences of 19 viruses belonging to 12 viral families were identified. Based on the established sequence identity thresholds (<80% nucleotide identity over the full genome or <90% amino acid identity in the RdRp domain), six viruses were proposed as novel viruses: Tick alpharicinrha-like virus isolate MTG (TARV), Tick pegi-like virus isolate MTG (TPV), Tick jingmen-like virus isolate MTG (TJMV), Tick noda-like virus isolate MTG (TNV), Tick tymo-like virus isolate MTG (TTV), and Tick tombus-like virus isolate MTG (TTBV). The number of viral species varied considerably across sampling site. Site 1 harbored the highest viral richness (seven species), followed by site 2 (six species), while site 3 yielded the fewest (two species). Notably, *Haemaphysalis japonica* was detected exclusively at Site 2, which is characterized by a rural forest ecosystem, suggesting a potential association between tick species composition and habitat complexity.

### 3.2 Phylogenetic analysis

#### 3.2.1 Phylogenetic analysis of *Flaviviridae*

**Fig. 2:**
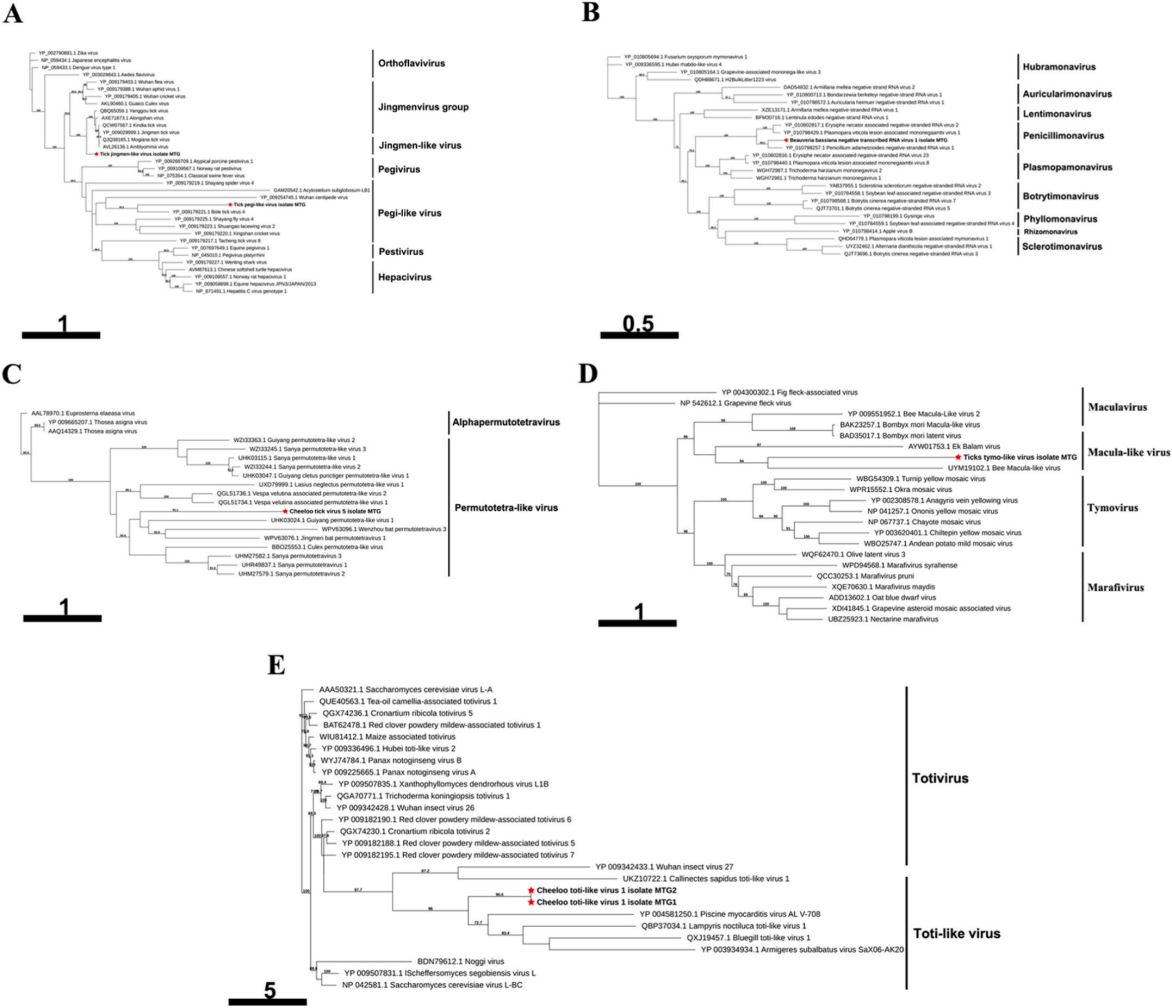
Phylogenetic tree of the Flaviviridae, Mymonaviridae, Permutotetraviridae, Tymovirales, and Orthototiviridae family based on the amino acid sequence of the ORF containing RdRp. (A) The phylogenetic tree of Flaviviridae. (B) The phylogenetic tree of Mymonaviridae. (C) The phylogenetic tree of Permutotetraviridae. (D) The phylogenetic tree of Tymovirales. The virus detected in this study is highlighted in bold white or black font for better visibility and mark it with Red five-pointed stars (★). Each virus genus is listed after the virus name.

The family *Flaviviridae* comprises numerous members, particularly within the genus *Flavivirus*, many of which are arthropod-borne and represent important human and veterinary pathogens, including the yellow fever virus, dengue virus, and West Nile virus [19]. In this study, two viruses belonging to the *Flaviviridae* were identified: TPV, which clusters within the *pegivirus* genus and TJMV, a member of the Jingmenvirus group. The amino acid identities of TPV with Bole tick virus 4 strain (Iftin/H.dromedarii/2018) were 67.58%, while the four fragments exhibited amino acid identities ranging from 26.44% to 52.80% compared to other Jingmen viruses (Table 1). Phylogenetic analysis revealed that TPV is closest related to Bole tick virus 4 and belongs to an unclassified genus in the *Flaviviridae* family (Fig. 2A). TJMV comprises four gene fragments (S1, S2, S3, and S4), encoding five proteins: NSP1(flavivirus NS5-like protein), NSP2 (flavivirus NS3-like protein), VP1, VP2, and VP3 and clusters phylogenetically with members of the Jingmenvirus group (Fig. 2A) [20]. Since the initial discovery of ingenious, members of this group have been identified in the blood of tick-bitten human patients, underscoring their potential threat to public health. The protein characteristics of the Jingmenvirus identified in this study warrant further investigation.

**Table 1:** Overview of viruses detected by Next-generation sequencing.

Classification	Virus (abbreviation)	Closet relative (% nt identity)	Genome (bp)
Hepelivirales	Hepelivirales sp.	95.60%-95.75%	5409/5425/5427
Mymonaviridae	*Beauveria bassiana* negative transcribed RNA virus 1	94.49%	6173
Nodaviridae	Cheeloo noda-like virus 3 (CNDV3)	99.09%	1668/1670
Permutotetraviridae	Cheeloo tick virus 5 (CTV5)	89.06%	4854
Phenuiviridae			
Uukuvirus	Cheeloo uukuvirus (CUV)	97.28%-99.86%	6514/1803/6570/1805
	Uukuvirus dabieshanense (UDH)	98.64%-98.69%	6530/1810/6527/1752
	Dabieshan Tick Virus (DTV)	98.63%-99.42%	6514/6495
Solemoviridae	Hubei sobemo-like virus 15 (HBSV15)	98.43%	2931
Rhabdoviridae			
Alpharicinrhavirus	Guyuan Rhabd tick virus 1 (GRTV1)	99.05%	11553/11552/11556
	Tahe rhabdovirus 1 (TRV1)	99.35%	11335
Tombusviridae			
Luteovirus.	Cheeloo tick virus 3 (CTV3)	99.64%-99.75%	3380/1609/3370/1609
Orthototiviridae	Cheeloo toti-like virus 1 (CTLV1)	96.04%-96.09%	7321/7319
Peribunyaviridae	Shanxi tick virus 2 (SXTV2)	96.65%-99.56%	10703/4442/1921/12077/12086/4433/1821

Classification	Virus (abbreviation)	Coding protein	Closet relative (% aa identity)	Genome (bp)
Rhabdoviridae	Tick alpharicinrha-like virus isolate MTG (TARV)	Polyprotein	Rhabdoviridae sp. isolate TIGMIC_1 (58.46%)	11002
Flaviviridae				
pegi-like virus	Tick pegi-like virus isolate MTG (TPV)	Polyprotein	Bole tick virus 4 strain Iftin/H.dromedarii/2018 (67.58%)	10982
Jingmenvirus group	Tick jingmen-like virus isolate MTG (TJMV)	NS5-like protein	Yanggou tick virus strain YG NS5-like protein gene (52.80%)	3141
		NS3-like protein	Mogiana tick virus isolate MGTV/V4/11 segment 3 (41.27%)	2938
		VP2-3	Mogiana tick virus isolate MGTV/V4/11 VP2-3 gene (44.10%)	2888
		glycoprotein	Jingmen tick virus isolate Kosovo 2013-17-266 segment 2 (26.44%)	2520
Nodaviridae	Tick noda-like virus isolate MTG (TNV)	RNA-dependent RNA polymerase	Nodaviridae sp. isolate Sarthropod030_noda1 (47.20%)	2726
Tymoviridae	Tick tymo-like virus isolate MTG (TTV)	polyprotein	Guarapuava tymovirus-like 1 isolate GTV-like1_3 (47.22%)	6506
Tombusviridae	Tick tombus-like virus isolate MTG (TTBV)	hypothetical protein 2	Hubei tombus-like virus 13 strain WHYY18977 (40.99%)	4185
Nodaviridae	Tick noda-like virus isolate MTG (TNV)	Polyprotein	Rhabdoviridae sp. isolate TIGMIC_1 (58.46%)	11002

#### 3.2.2 Phylogenetic analysis of *Mymonaviridae*

Members of the family *Mymonaviridae* typically infect filamentous fungi, although they have also been found in metagenomic studies of insects, oomycetes, and plants [21]. In this study, a virus named *Beauveria basiana* negative transcribed RNA virus 1 was identified. Currently, only one strain has been recorded in the GenBank database, which was discovered in Jilin in 2023 (GenBank: OR737625.1) (Table 1). Phylogenetically, this virus clusters within the same evolutionary branch as members of *Penicilliimonavirus* (Fig. 2B).

#### 3.2.3 Phylogenetic analysis of *Permutotetraviridae*

The family *Permutotetraviridae,* established in 2012, contains a single genus: *Alphapermutotetravirus* [22]. A strain of Cheeloo tick virus 5 was identified in our tick's samples, sharing 89.06% nucleotide identity with Cheeloo tick virus 5 isolate CLCM-135 (GenBank: OR148425.1). In the evolutionary, it belongs to the Permutotetra-like virus branch (Fig. 2C).

#### 3.2.4 Phylogenetic analysis of *Tymoviridae*

Members of the family *Tymoviridae* primarily infect dicotyledonous plants, and the host range of individual viral species is very narrow, sometimes limited to a single type of host [23]. A tymo-like virus, tentatively named Tick tymo-like virus, belonging to the family Tymoviridae was identified in our samples (Fig. 2D). It showed amino acid identities of 47.22% with Guarapuava tymovirus-like 1 isolate GTV-like1_3 (GenBank: AYV61001.1) (Table 1).

#### 3.2.5 Phylogenetic analysis of *Orthototiviridae*

The family *Orthototiviridae,* established in 2023, encompasses dsRNA viruses infecting protists, fungi, plants, and invertebrates [24]. In this study, two viral strains were identified, sharing 96.04%–96.09% nucleotide identity with Cheeloo toti-like virus 1 CLCM-061 (GenBank: OR148387.1). And clustering with another toti-like virus in phylogenetic tree (Fig. 2E).

#### 3.2.6 Phylogenetic analysis of *Hepelivirales*

**Fig. 3:**
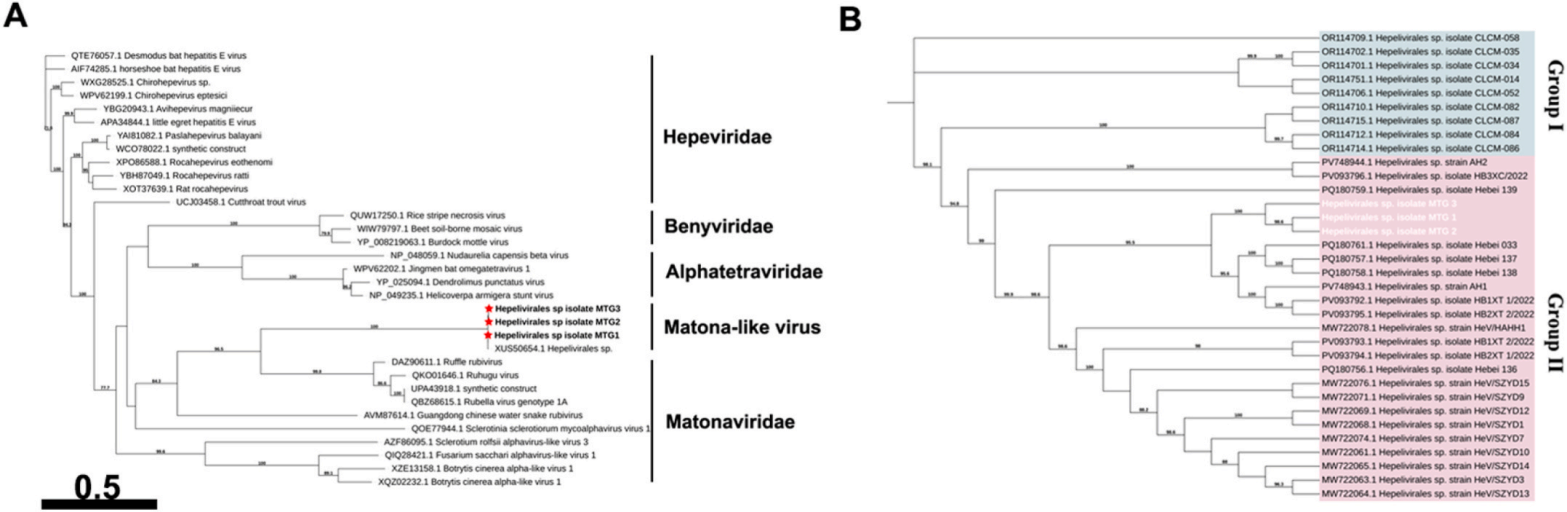
Phylogenetic tree of the Hepelivirales. (A) The phylogenetic tree of Hepelivirales based on the amino acid sequence of the ORF containing RdRp. (B) The phylogenetic tree of Hepelivirales sp. based on the nucleic acid sequence. The virus detected in this study is highlighted in bold white or black font for better visibility and mark it with Red five-pointed stars (★). Each virus genus is listed after the virus name.

In this study, we identified three strains belonging to the *Hepelivirales*, which shared 95.60%-95.74% nucleotide identity with Hepelivirales sp. isolate Hebei_100 (GenBank: PQ180745.1). The three-strain clustered together with Matona-like virus (Fig. 3A). The phylogenetic tree based on the whole-genome sequence showed that our isolates clustered with strains previously discovered in Hebei, China (Fig. 3B).

#### 3.2.7 Phylogenetic analysis of *Nodaviridae*

**Fig. 4:**
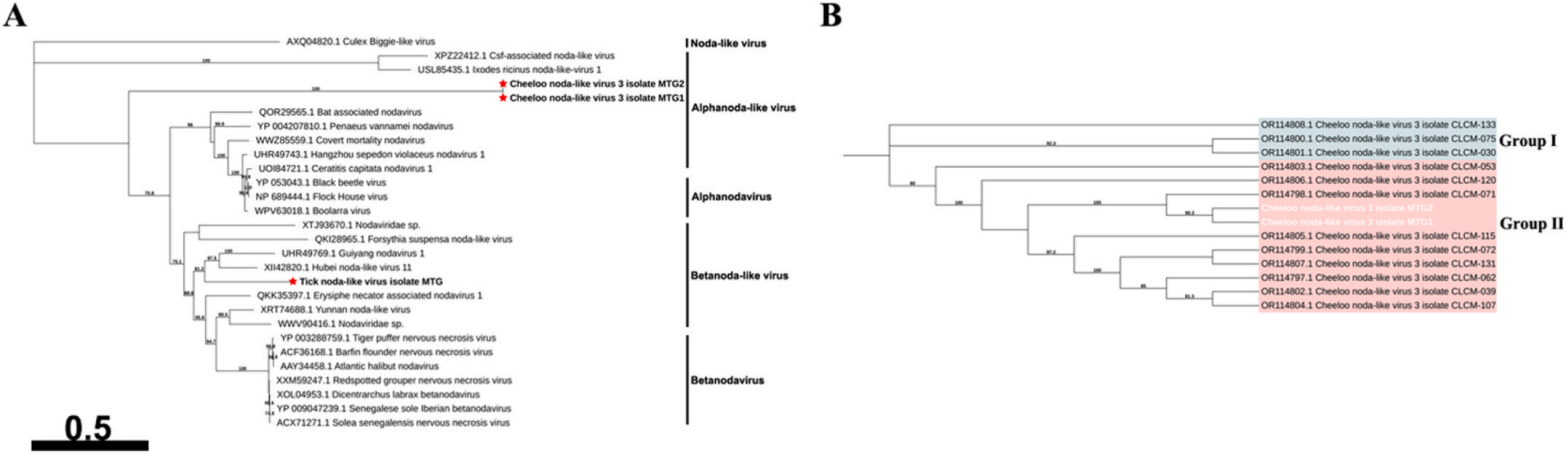
Phylogenetic analyze of the Nodaviridae. (A) The phylogenetic tree of Nodaviridae based on the amino acid sequence of the ORF containing RdRp. (B) The phylogenetic tree of Cheeloo noda-like virus 3 based on the nucleic acid sequence. The virus detected in this study is highlighted in bold white or black font for better visibility and mark it with Red five-pointed stars (★). Each virus genus is listed after the virus name.

The family *Nodaviridae* includes two genera, *Alphanodaviruses*, which infects insects whereas *Betanodaviruses*, which infects fish [25]. Two viruses belonging to the *Nodaviridae* were identified in tick samples, including two unclassified viruses, TNV and Cheeloo noda-like virus 3 (CNV3). The nucleotide identity of the two CNV3s identified in different samples with other viruses is 99.09%. Currently, CNV3 has only been detected in Shandong, China. Additionally, a Betanoda-like virus, Tick noda-like virus, exhibited 47.20% amino acid identity with Nodaviridae sp. isolate Sarthropod030_noda1 (GenBank: XNT20824.1) (Fig. 4A). The phylogenetic tree based on the nucleotide sequence of CNV3s showed that our strains clustered with the isolates identified in Shandong (Fig. 4B).

#### 3.2.8 Phylogenetic analysis of *Phenuiviridae*

**Fig. 5:**
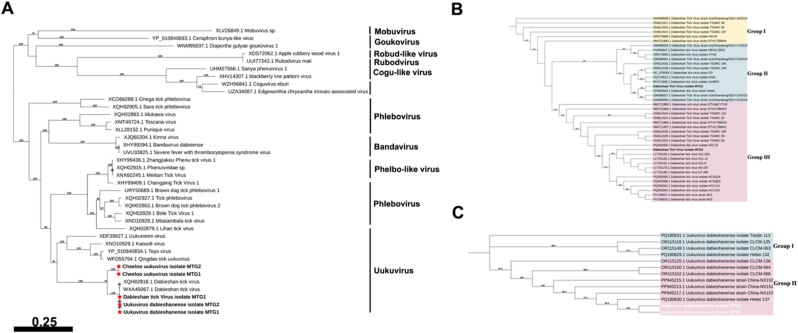
Phylogenetic analyze of the Phenuiviridae. (A) The phylogenetic tree of Phenuiviridae based on the amino acid sequence of the ORF containing RdRp. (B) The phylogenetic tree of Dabieshan tick virus based on the nucleic acid sequence. (C) The phylogenetic tree of Uukuvirus dabieshanense based on the nucleic acid sequence. TThe virus detected in this study is highlighted in bold white or black font for better visibility and mark it with Red five-pointed stars (★). Each virus genus is listed after the virus name.

The genus *Uukuvirus,* belonging to the family *Phenuiviridae,* comprises 23 viruses that infect mammals and birds and are transmitted by ticks [26]. The three identified viruses in our tick samples belong to the genus *Uukuvirus*, including Cheeloo uukuvirus (CUV), Uukuvirus dabieshanense (UDH), and Dabieshan tick virus (DTV) (Fig. 5A) in this study. The nucleotide identities of these viruses with other known strains were 96.79%-99.86%, 98.64%-98.69%, and 98.63%-99.42%, respectively. Phylogenetic analysis of nucleic acid sequences showed two DTV strains clustered within the same evolutionary branch as the strains found in Shandong, China and Japan (Fig. 5B), while the two UDH strains formed a clade most closely related to the strain identified in Hebei, China (Fig. 5C).

#### 3.2.9 Phylogenetic analysis of *Solemoviridae*

**Fig. 6:**
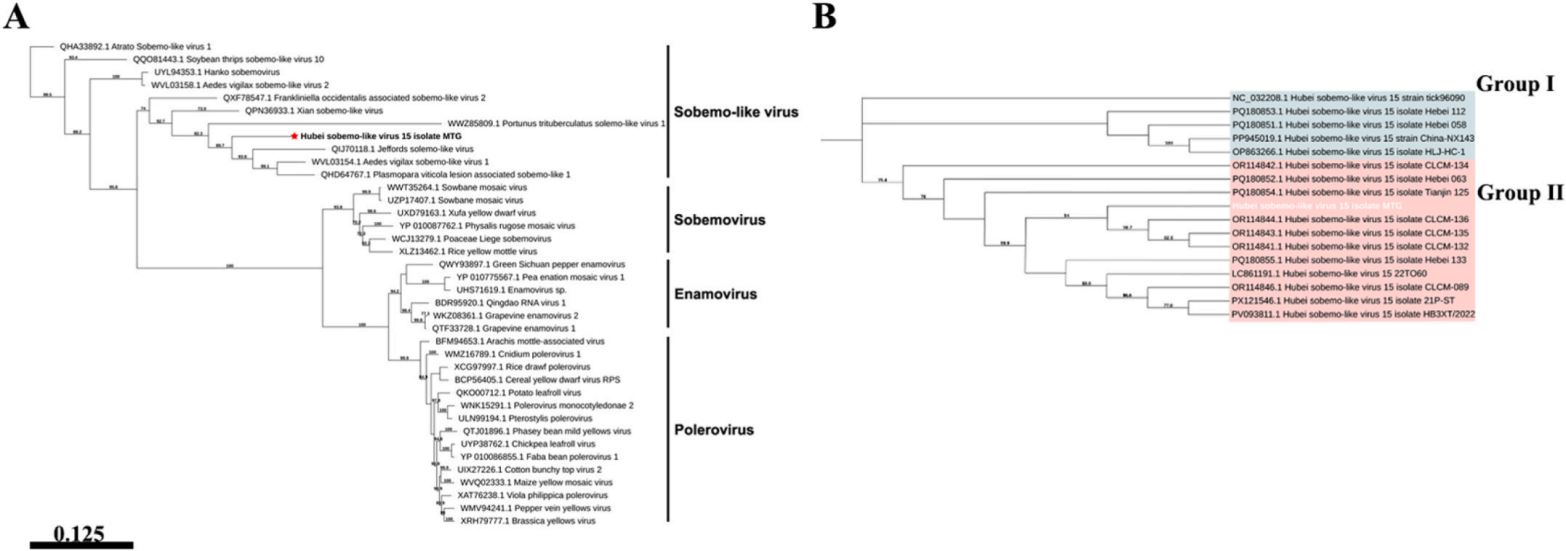
Phylogenetic analyze of the Solemoviridae. (A) The phylogenetic tree of Solemoviridae based on the amino acid sequence of the ORF containing RdRp. (B) The phylogenetic tree of Hubei sobemo-like virus 15 based on the nucleic acid sequence. The virus detected in this study is highlighted in bold white or black font for better visibility and mark it with Red five-pointed stars (★). Each virus genus is listed after the virus name.

The family *Solemoviridae* includes important pathogens in many major crops, which are readily transmitted mechanically and also transmitted by insects [27]. In this study, we identified Hubei sobemo-like virus 15 (HSMV15). This virus exhibited the highest nucleotide identity with the strain identified in Shandong in 2024. Consistently, phylogenetic analysis showed that HSMV15 was also closely related to the strains identified in Shandong, China (Fig. 6B). HSMVV15 could not be clearly assigned to any established genus within the *Solemoviridae* (Fig. 6A).

#### 3.2.10 Phylogenetic analysis of *Rhabdoviridae*

**Fig. 7:**
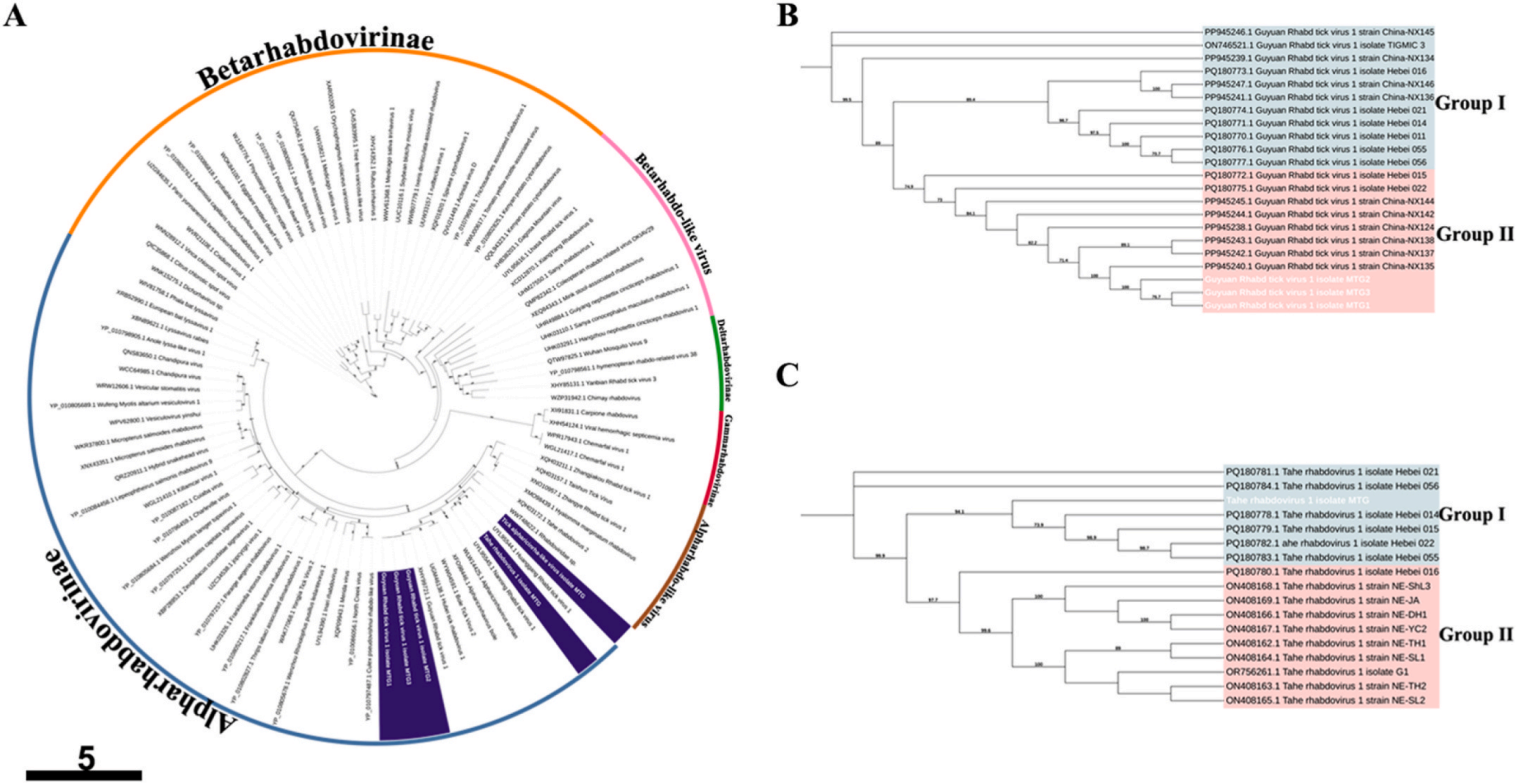
Phylogenetic analyze of the Rhabdoviridae. (A) The phylogenetic tree of Rhabdoviridae based on the amino acid sequence of the ORF containing RdRp. (B) The phylogenetic tree of Guyuan Rhabd tick virus 1 based on the nucleic acid sequence. (C) The phylogenetic tree of Tahe rhabdovirus 1 based on the nucleic acid sequence. The virus detected in this study is highlighted in bold white or black font for better visibility and mark it with Red five-pointed stars (★). Each virus genus is listed after the virus name.

The family *Rhabdoviridae* is highly diverse, comprising 434 viruses, many of which are important pathogens of humans, livestock, fish, or crops [28]. Invertebrates can serve as either unique hosts or biological vectors. Several viruses belonging to the *Alpharicinrhavirus* were identified, named GRTV1 and TRV1 (Fig. 7A). The nucleotide sequences of GRTV1 and TRV1 shared 99.05% and 99.35% identity with other strains, respectively. GRTV1 and TRV1 were clustered with strains from Ningxia and Hebei in the phylogenetic tree (Fig. 7B and C). A virus belonging to the Alpharicinrhavirus genus, exhibiting 58.46% amino acid identity of with the RdRp of Rhabdoviridae sp. isolate TIGMIC_1, was identified and tentatively named as Tick alpharicinrha-like virus (TARV) (Fig. 7A).

#### 3.2.11 Phylogenetic analysis of *Tolivirales*

**Fig. 8:**
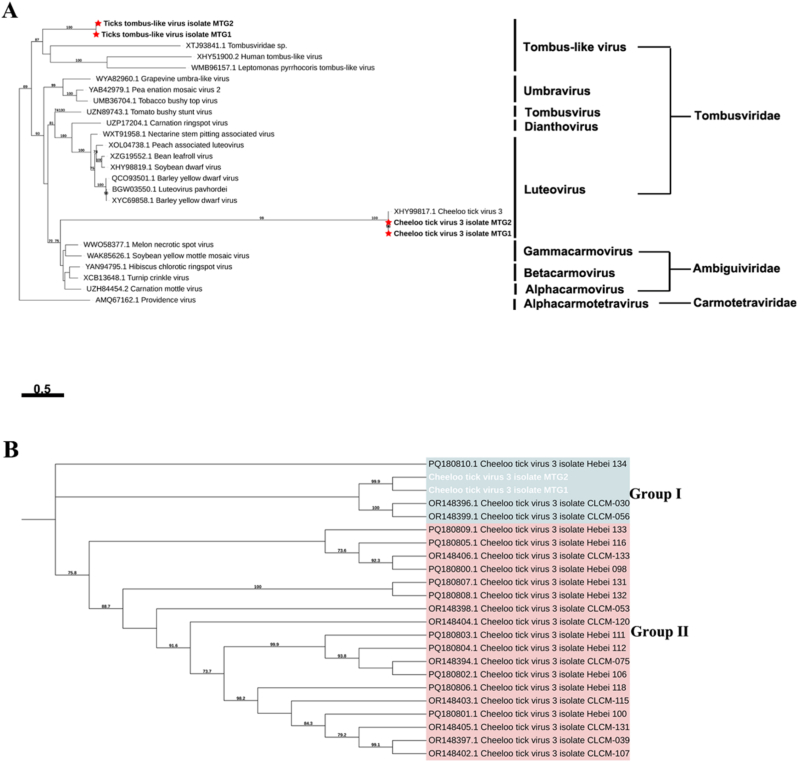
Phylogenetic analyze of the Toliovirales. (A) The phylogenetic tree of Tombusviridae based on the amino acid sequence of the ORF containing RdRp. (B) The phylogenetic tree of Cheeloo tick virus 3 based on the nucleic acid sequence. The virus detected in this study is highlighted in bold white or black font for better visibility and mark it with Red five-pointed stars (★). Each virus genus is listed after the virus name.

Conserved domain prediction revealed that the RNA-dependent RNA polymerase (RdRp) sequences of two viruses belong to the order *Tolivirales*. Phylogenetic analysis showed that these two viruses clustered with members of the family *Tombusviridae*; they were herefore tentatively named as tick tombus-like virus (TTV) (Fig. 8A). In addition, two strains of Cheeloo tick virus 3 were identified, sharing 99.64% −99.75% nucleotide identity with other known strains. These strains clustered within the same evolutionary branch as other members of *Tombusviridae* (Fig. 8A).

### 3.3 Protein characteristics of TJMV

**Fig. 9:**
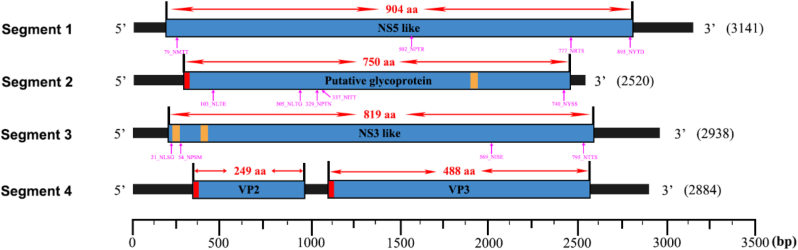
Schematic diagram of the genome and functional regions of various proteins of TJMV. The predicted ORF is shown in a dark blue box, the signal peptide is shown in a red box, the transmembrane region is shown in a yellow box, and the N-linked glycosylation site is shown in a pink arrow.

The TJMV genome comprised segments 1 to 4 based on BLAST and CD-search results. Although this protein lacks some enzyme cleavage sites present in other Jingmenviruses (Fig. 9, Fig. S1), it features four predicted N-linked glycosylation sites. Segment 1 encodes an NS5-like protein with typical RNA-directed RNA polymerase and FtsJ-like methyltransferase conserved motifs, although it lacks some enzyme cleavage sites compared to other Jingmenviruses (Fig. 9, Fig. S1). Segment 3 encodes the non-structural protein NS3, resembling the NS2b-NS3 complex of flaviviruses. This NS3-like protein contains a signal peptide region and two transmembrane regions, with a predicted membrane-binding region located between residues 50-115 (Fig. 9). Notably, we identified a binding pocket on the TJMV NS3-like protein structurally analogous to the site that interacting with the small molecule inhibitor MI-2258 in Zika virus (Fig. S2). MI-2258, synthesized by modifying the side chains of glycine and lysine residues (reported as V7T-GLY-V8N-LYS-4J5), is known to interfere with viral replication by inhibiting protease activity [29]. However, our molecular docking analysis revealed key amino acid substitutions within this pocket (e.g., TRP50 and TYR150 in 8AQK replaced by PHE50 and PHE103 in TJMV NS3), suggesting altered binding affinities and pocket dimensions.

Segment 2 and Segment 4 encode three structural proteins: glycoprotein, VP2, and VP3 (Fig. 9). The glycoprotein possesses a signal peptide and a C-terminal transmembrane region at position 543, along with potential N-glycosylation sites at residues 103, 305, 329, 337, and 740. However, it lacks the typical fusion loop and domain III found in similar TJMV, which are associated with virus–cell binding and viral entry. Segment 4 encodes VP2 and VP3, which have no predicted transmembrane regions or N-linked glycosylation sites except for signal peptidase recognition sites (Fig. 9).

Crucially, while the phylogenetic relatedness and structural features described above strongly suggest a potential zoonotic capacity of TJMV, they do not constitute direct evidence of human infection or pathogenicity. To further explore this theoretical immunogenic potential, we computationally predicted T-cell (NetMHCIIpan) and B-cell (ABCpred) epitopes of the TJMV glycoprotein (Table S3, Table S4). The identification of multiple strong MHC-II binding peptides (%Rank <1%) and high-scoring linear B-cell epitopes (scores ≥0.90) implies that the viral surface glycoprotein possesses a theoretical signature capable of eliciting human adaptive immune responses. Nevertheless, we strictly emphasize that the suspected human pathogenicity of TJMV remains hypothetical. To firmly establish its actual public health relevance, future verification is indispensable.

## 4 Discussion

In this study, we characterized the diverse RNA virome found in ticks collected from Mentougou, Beijing, China. We identified a total of 19 distinct viral species across 12 viral families, including *Hepelivirales, Mymonaviridae, Phenuiviridae, Permutotetraviridae, Rhabdoviridae, Tombusviridae, Totiviridae, Nodaviridae, Flaviviridae, Tymoviridae, Solemoviridae*, and *Peribunyaviridae*. Among these 19 viruses, we identified both animal and plant viruses, including six novel viruses, highlighting the considerable diversity and complexity of the tick-associated viral community. Overall, this study fills a significant data gap for this ecological region and provides essential baseline information for evaluating the potential risk of emerging infectious diseases in the Beijing area. To explore the ecological drivers underlying this viral diversity, we compared virus distribution across three sampling sites with distinct habitat types. Although the three sites in this study are located within a relatively small geographical area, they exhibited marked differences in vegetation structure, tick species composition, and viral diversity. Viral richness increased along a gradient of habitat complexity, from urban residential areas to urban parks and then to rural forests, indicating that structurally more complex natural vegetation supports greater viral diversity. This may be attributed to the provision of diverse microhabitats and vertebrate host communities, which facilitate tick-host-virus interactions. Notably, *Haemaphysalis japonica* was detected exclusively at Site 1 (rural forest),co-occurring with the dominant species *H. longicornis*; however, this site yielded fewer viruses than Site 3, where only *H. longicornis* was present. This observation indicates that tick species richness alone does not necessarily determine virome diversity, and that ecological context—particularly vegetation structure and host community composition—may exert a stronger influence. The precise geographic coordinates provided in this study will enable future comparative and meta-analytic investigations to assess spatial patterns of tick-borne viromes across Beijing and other temperate regions, underscoring the importance of high-resolution georeferencing and ecological characterization in tick surveillance programs.

Among the identified viruses, several are commonly reported in tick virome studies, such as Cheeloo uukuvirus (CUV), Uukuvirus dabieshanense (UDH), and Dabieshan tick virus (DTV) of the family *Phenuiviridae*, as well as Shanxi tick virus 2 (SXTV2) of the family *Peribunyaviridae*. Although no human infection with these viruses have been documented to date, laboratory studies have shown that *Hepelivirales sp.* can replicate in Vero cells without causing apparent cytopathic effects (CPE). Notably, recent GenBank database records indicate that genetic segments of SXTV2 and DTV have been detected in *Homo sapiens* samples (e.g. PV018763.1 and PV014884.1), suggesting that they may pose a risk to public health. We also identified several rarely reported viruses, including *Beauveria bassiana* negative transcribed RNA virus 1, Cheeloo tick virus 5, and Cheeloo toti-like virus 1, all of which have only recently been documented in ticks. The detection of these viruses not only validates their sequence authenticity but also underscores their broad distribution in tick populations. With the increasing application of metatranscriptomic sequencing in tick virome research, a growing number of novel viruses continue to be identified [30,31]. Repeated detection across independent studies will further elucidate their ecological distribution and transmission dynamics [32].

Additionally, this study discovered six novel viruses belonging to five families: *Rhabdoviridae, Flaviviridae, Tymoviridae, Tombusviridae,* and *Nodaviridae*. Among these, members of the *Flaviviridae* and *Nodaviridae* are considered to pose higher pathogenicity risks to humans and mammals. Segmented *Flaviviridae* has recently emerged as notable tick-borne virus, with reported from Brazil, Kosovo, Uganda, Türkiye, French Antilles and Trinidad and Tobago [33]. JMTV was first identified in 2014 in *Rhipicephalus microplus* ticks sampled from Jingmenin Hubei and Wenzhou in Zhejiang, China [34]. Beyond ticks and mosquitoes, JMTV has also been detected in cattle and humans, and recent studies have linked it to febrile illnesses, indicating its potential to infect a broad range of mammals v [35,36]. The Jingmen-like virus identified in this study exhibited low amino acid identity with other known Jingmen-viruses. While the non-structural proteins of JMTV contain conserved motifs typical of flaviviral RdRP and helicase, its structural proteins show no significant sequence or structural homology to any known viral proteins. This may reflect rapid structural protein evolution, or the corresponding homologous sequences may remain undiscovered [34]. Some studies suggest that discontinuous genome assembly or erroneous recombination may lead to the inclusion of novel segments in the viral genome [34,37]. We predicted potential B-cell and T-cell epitopes of TJMV glycoproteins, However, we strictly emphasize that the suspected human pathogenicity of TJMV remains hypothetical. To accurately determine its actual public health relevance, future verification is essential. This includes: (i) serological monitoring (such as ELISA) to screen for specific antibodies against TJMV in high-risk populations; (ii) molecular testing (such as qPCR or metagenomic sequencing) in patients with corresponding febrile illnesses; and (iii) functional *in vitro* assays to evaluate viral entry into human cells, as well as *in vivo* animal infection models to verify pathogenicity. Another flavivirus identified, TPV, clusters with pegiviruses, which are primarily found in mammals, suggesting a possible zoonotic risk [38,39].

Several plant-associated viruses were also detected, including Hubei Solemoviridae sobemo like virus 15(*Solemoviridae*), TTV (*Tymoviridae*), and TTBV(*Tombusviridae*). Compared to known pathogenic tick-borne virus in families such as *Phenuiviridae*and *Flaviviridae*, these plant-infecting viruses are less likely to cause disease in mammals.

## 5 Conclusions

In conclusion, this study provides the first virome profile of ticks in Mentougou, Beijing, identifying 29 strains of 19 viruses from 12 families via metatranscriptomic sequencing. Six novel viruses-TARV, TPV, TTV, TTBV, TJMV, and TNV-were reported, substantially expanding our understanding of viral diversity in this region. However, several limitations should be acknowledged. Although multiple novel tick-borne viruses were detected, previously documented pathogenic viruses in Beijing were not identified in this study. This absence may be attributed to multiple factors, including the limited geographical scope of sampling, the seasonal timing of collection (July, which may not coincide with peak circulation of certain pathogens), methodological constraints (e.g., the use of metatranscriptomic sequencing without viral enrichment, which reduces sensitivity for low-titer viruses), and ecological variables such as local host community composition and tick population densities. Future research should expand the geographical and taxonomic scope of tick collection to better understand viral diversity and host–virus ecological relationships.

## Ethics approval and consent to participate

Not applicable.

## Data availability statement

Nucleic acid sequence are deposited in National Microbiology Data Center (NMDC) with accession numbers NMDCN0009VGH, NMDCN0009VGI, NMDCN0009VGJ, NMDCN0009VGK, NMDCN0009VGL, NMDCN0009VGM, NMDCN0009VGN, NMDCN0009VGO, NMDCN0009VGP, NMDCN0009VGQ, NMDCN0009VGR, NMDCN0009VGS, NMDCN0009VGT, NMDCN0009VGU, NMDCN0009VGV, NMDCN0009VH0, NMDCN0009VH1, NMDCN0009VH2, NMDCN0009VH3, NMDCN0009VH4, NMDCN0009VH5, NMDCN0009VH6, NMDCN0009VH7, NMDCN0009VH8,NMDCN0009VH9,NMDCN0009VHA,NMDCN0009VHB,NMDCN0009VHC,NMDCN0009VHD,NMDCN0009VHE,NMDCN0009VHF,NMDCN0009VHG,NMDCN0009VHH,NMDCN0009VHI,NMDCN0009VHJ,NMDCN0009VHK,NMDCN0009VHL,NMDCN0009VHM,NMDCN0009VHN,NMDCN0009VHO,NMDCN0009VHP,NMDCN0009VHQ,NMDCN0009VHR,NMDCN0009VHS. Raw sequencing data are deposited in NMDC with accession numbers NMDC40097794, NMDC40097795, NMDC40097796, NMDC40097797, NMDC40097798, NMDC40097799, NMDC40097800, NMDC40097801. Meanwhile, the metagenomic data are available in the NCBI Sequence Read Archive (SRA) (SRR37116342-SRR37116349) under Bioproject PRJNA1418940, and the viral assembled genomes are available in the GenBank with the accession numbers PZ065977-PZ066020.

## Consent for publication

The manuscript is original and has not been published elsewhere. All authors agree to the submission and have contributed to the work. There are no conflicts of interest.

## Funding statement

This study was supported by the National Science and Technology Major Project of China (No. 2025ZD01900105), the 10.13039/501100012166National Key R&D Program of China (No. 2023YFC2308204 and No. 2022YFC2602402), the Project of the National Pathogen Resource Collection Center (No. NPRC-32), the SKLID Development Grant (No. 2011SKLID104).

## CRediT authorship contribution statement

**Qiangqiang Shi:** Conceptualization, Data curation, Formal analysis. **Qinqin Song:** Data curation, Formal analysis, Software. **Xuan Liu:** Data curation. **Guoyong Mei:** Supervision, Writing – review & editing. **Chen Gao:** Formal analysis, Software. **Haijun Du:** Formal analysis, Methodology. **Zhiqiang Xia:** Software. **Mi Liu:** Resources, Supervision. **Juan Song:** Data curation, Resources. **Lifeng Zhang:** Investigation. **Rundong Zhu:** Investigation. **Ze Cheng:** Methodology. **Jin Cao:** Software. **Duo Rao:** Software. **Yuqin Zhang:** Formal analysis. **Zhiyue Wang:** Investigation, Resources, Writing – review & editing. **Jun Han:** Data curation, Formal analysis, Funding acquisition, Writing – review & editing.

## Declaration of competing interest

The authors declare that they have no known competing financial interests or personal relationships that could have appeared to influence the work reported in this paper.
