# Supplementary material for: Macrogenomic analysis showcases the diversity of tick RNA viruses in Mentougou, Beijing, China
Source: New Microbes New Infect. 2026 Jul 24;73:101819. doi: 10.1016/j.nmni.2026.101819 (PMC13449411; doi:10.1016/j.nmni.2026.101819)
Supplement: Supplementary file 1 [file mmc1.docx]

Figure S1: Amino acid alignment of the NS5 proteins of TJMV and representative Jingmen virus group members. This displays some conservative motifs predicted in interPro. HMV: Harz mountain virus, YGTV: Yanggou tick virus, XJTV1: Xinjiang tick virus 1, BSFTV: Baishan forest tick virus, TKV: Takachi virus, JMTV: Jingmen tick virus, GDJMV: Guangdong Jingmen-like virus, TJMV: Ticks jingmen-like virus.

Figure S2: Amino acid alignment of the NS3 proteins of TJMV and representative Jingmen virus group members. This displays some conservative motifs predicted in interPro. HMV: Harz mountain virus, YGTV: Yanggou tick virus, XJTV1: Xinjiang tick virus 1, BSFTV: Baishan forest tick virus, TKV: Takachi virus, JMTV: Jingmen tick virus, GDJMV: Guangdong Jingmen-like virus, TJMV: Ticks jingmen-like virus.

Figure S3: Amino acid alignment of the glycoprotein of TJMV and JMTV. From top to bottom, there are four sequences. The first three are the amino acid sequences and glycoprotein sequences of JMTV published at different times. YEJ22930.1 represents the glycoprotein amino acid sequence of TJMV.

| **Description** | **Position** |
| --- | --- |
| **NS5 like protein** |  |
| Signal peptide | None |
| Transmembrane | None |
| N-linked glycosylation | 79 NMTT |
|  | 502 NPTR |
|  | 777 NRTS |
|  | 895 NYTD |
| **NS3 like protein** |  |
| Signal peptide | 1-18 |
| Transmembrane | 22-44 |
|  | 116 -138 |
| N-linked glycosylation | 21_NLSG |
|  | 54_NPSM |
|  | 569_NISE |
|  | 795_NTTS |
| **Putative glycoprotein** |  |
| Signal peptide | 1-18 |
| Transmembrane | 543-565 |
| N-linked glycosylation | 103_NLTE |
|  | 305_NLTG |
|  | 329_NPTN |
|  | 337_NITT |
|  | 740_NYSS |
| **VP2** |  |
| Signal peptide | 1-18 |
| Transmembrane | None |
| N-linked glycosylation | None |
| **VP3** |  |
| Signal peptide | 1-18 |
| Transmembrane | None |
| N-linked glycosylation | None |
|  |  |

Table S2: Signal peptide, transmembrane region, and glycosylation site prediction details of TJMV.

Table S3：Predicted docking interfaces of IM-2258

| **Target Protein** | **Interacting Residues (Binding Interface)** |
| --- | --- |
| NS3 protein of ZIKV (PDB ID: 8AQK) | **VAL36, TRP50, HIS51, VAL52, THR53, LYS54, VAL72, LYS73, GLN74, ASP75, PHE116, ILE123, LEU128, ASP129, TYR130, PRO131, ALA132, GLY133, THR134, SER135, TYR150, GLY151, ASN152, GLY153, VAL154, VAL155, GLY159, SER160, TYR161, ALA164, THR166**. |
| NS3 protein of TJMV | **SER29, SER30, PHE50, SER52, PRO53, THR54, ALA55, ASP56, PHE57, THR63, PRO64, VAL65, VAL66, GLU67, ARG68, PRO69, GLY70, CYS94, ASN96, GLY99, HIS100, THR101, LEU102, PHE103, TYR117, ALA133, GLY134, THR135, ILE136, GLY137, ARG138, SER139, VAL140, GLU141**. |

Table S4: Predicted strong-binding T-cell epitopes of the glycoprotein of TJMV

| **Position** | **Peptide (15-mer)** | **Core binding motif (9-mer)** | **%Rank_EL** | **Bind Level** |
| --- | --- | --- | --- | --- |
| 474 | TLLYLAIVASPVMSA | YLAIVASPV | 0.42 | SB |
| 293 | ISDYTKVHGITANLT | YTKVHGITA | 0.55 | SB |
| 584 | FPGLLPLLAHHVFNN | LLPLLAHHV | 0.57 | SB |
| 473 | VTLLYLAIVASPVMS | YLAIVASPV | 0.65 | SB |
| 292 | PISDYTKVHGITANL | YTKVHGITA | 0.75 | SB |
| 708 | AKDLRYLRANLPPIT | LRYLRANLP | 0.77 | SB |
| 630 | ARRFLFLRDFGLQYH | FLFLRDFGL | 0.78 | SB |
| 583 | VFPGLLPLLAHHVFN | LLPLLAHHV | 0.92 | SB |
| 707 | SAKDLRYLRANLPPI | LRYLRANLP | 0.98 | SB |

Note: SB = Strong Binder, threshold < 1%

Table S5: Predicted high-scoring linear B-cell epitopes of the glycoprotein of TJMV

| **Rank** | **Start Position** | **Peptide Sequence (16-mer)** | **Prediction Score** |
| --- | --- | --- | --- |
| 1 | 340 | RHNITTPPGSVPLCGP | 0.95 |
| 1 | 234 | PHPQPTTTPYGSFCDT | 0.95 |
| 2 | 710 | EMSAKDLRYLRANLPP | 0.93 |
| 3 | 37 | PGSQDLSQPPRKVSRP | 0.92 |
| 3 | 294 | PGTPISDYTKVHGITA | 0.92 |
| 3 | 260 | HGNIFVDVTMTKVLTT | 0.92 |
| 3 | 199 | YRTQESYGSKVAWVTK | 0.92 |
| 3 | 154 | AHKIYFPPEYVTRTEV | 0.92 |
| 4 | 219 | TLNPKAPRLATVEVQP | 0.91 |

Note: only the top candidate epitopes with scores ≥ 0.90 are displayed.
